# Oligodontia and Facial Phenotype Associated with a Rare Syndrome

**DOI:** 10.1155/2022/1045327

**Published:** 2022-12-26

**Authors:** Fatima Ezzahra Zidane, Mustapha El Alloussi

**Affiliations:** College of Health Sciences, International Faculty of Dental Medicine, BioMed Unit, International University of Rabat, Technopolis Parc, Rocade of Rabat-Salé, Sala-Al Jadida 11100, Morocco

## Abstract

**Introduction:**

Oligodontia is a dental abnormality in which the patient is missing teeth. It is a hereditary disorder characterized by agenesis of more than six primary or permanent teeth, excluding the wisdom teeth. Oligodontia is often related with an abnormal size of teeth, conical shape, taurodontism, frequent enamel abnormalities, and delayed eruption. Oligodontia may be clinically isolated or associated with ectodermal dysplasia, a large group of rare diseases, and other syndromes. *Patient Information.* Dental characteristics of a six-and-a-half-year-old Moroccan boy with oligodontia and in apparent good health were described. *Clinical Findings.* Three syndromes associated with oligodontia have been discussed. Above all, based on the facial phenotype, Dubowitz syndrome has been retained as the most likely diagnostic hypothesis. This case could be the first reported case described in Morocco, but a thorough examination with genetic analysis must be carried out.

**Conclusion:**

Oligodontia could clinically be isolated or associated with ectodermal dysplasia, a large group of rare diseases, and other syndromes.

## 1. Introduction

Agenesis of teeth is the most common developmental dental anomaly in human beings. Several terms have been used to describe the congenital absence of teeth. Oligodontia implies agenesis of at least six definitive teeth, excluding wisdom teeth. Often teeth absent are the terminal teeth of a series [premolars (32–15%), maxillary lateral incisors (27%), and third molars (25%)] [1]. Oligodontia is a rare anomaly with a prevalence of 0.3% in permanent teeth and less in the primary dentition. The pathology can be isolated or can be associated with other ectodermal abnormalities and syndromes, such as ectodermal dysplasia, cleft lip, and palate [2]. Moreover, different epidemiological studies have shown a varying prevalence of oligodontia, depending on ethnic origin, with an average ranging from 0.1 to 0.2% [3]. Besides agenesis, other anomalies of size and shape can also be observed, such as rotation of the teeth, delayed dental development, and eruption [4]. Furthermore, hypo-development of the jaws and alveolar bone with modification of the facial structures and functional consequences were noticed. Optimally, agenesis should be detected earlier during childhood. Multiple dental agenesis is most frequently discovered fortuitously. The diagnosis can only be confirmed by the orthopantogram (the absence of the dental follicle is a sign of agenesis). Oligodontia is most frequently associated with syndromes and may be one of the signs to enhance the early diagnosis of these syndromes. The aim of this report was to describe the dental characteristics of a six-and-a-half-year-old Moroccan boy with oligodontia; this case might be the first reported case of Dubowitz syndrome (DS) described in Morocco, but a thorough examination with genetic analysis must be carried out.

## 2. Patient Information

A six-and-a-half-year-old Moroccan boy presented to the dental office for pain as a reason for consultation. The diagnosis of oligodontia has not been made previously.

## 3. Clinical Findings

**Figure 1:**
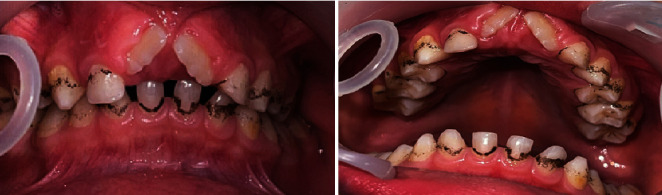
Chromogenic bacteria, rotation of the lower incisors, and a high narrow palate.

During the dental examination, the patient had a high-pitched voice and mild mental retardation (no test was assessed to quantify it). In the extraoral examination, clinical anomalies including a triangular face, small head, little frontal bossing, low-set ears, saddle nose, and eczema were found. The intraoral examination revealed mixed dentition, chromogenic bacteria, rotation of the upper incisors, and a high narrow palate (Figure 1).

**Figure 2:**
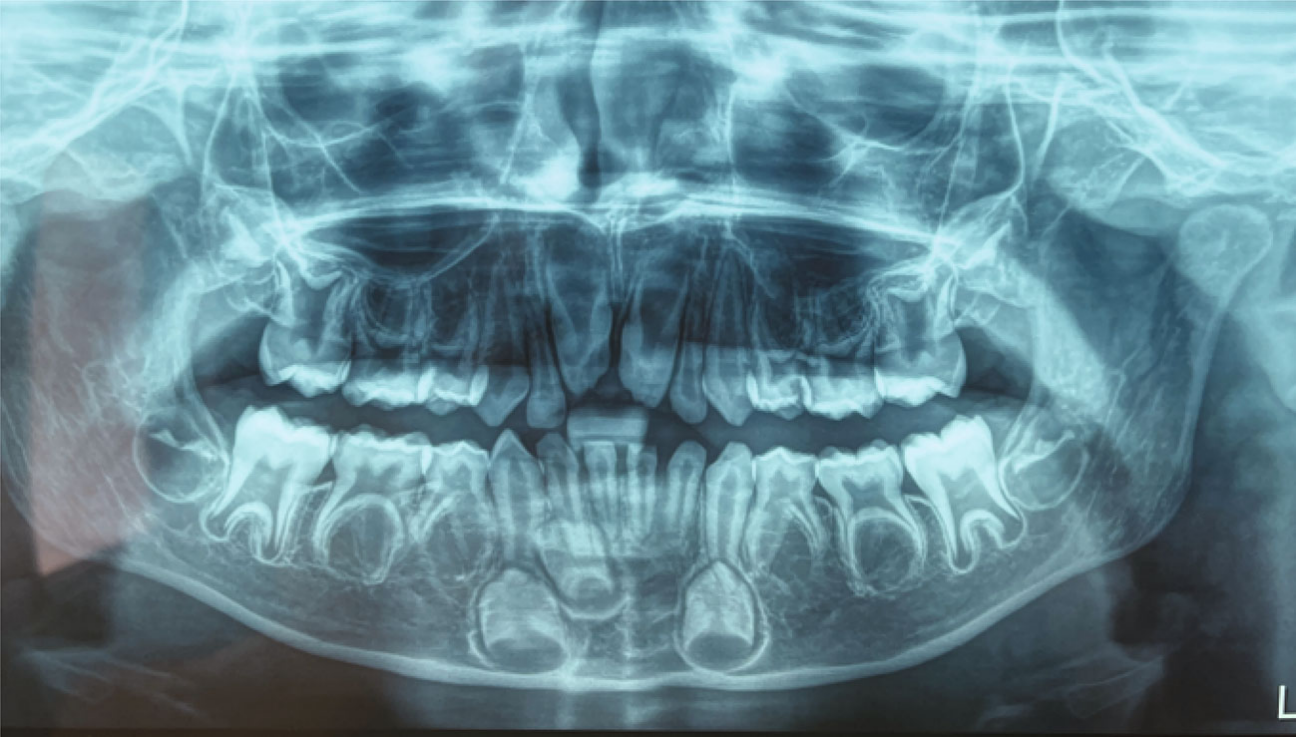
Panoramic radiographic view demonstrating oligodontia, chronologically delayed eruption, and taurodontism.

**Figure 3:**
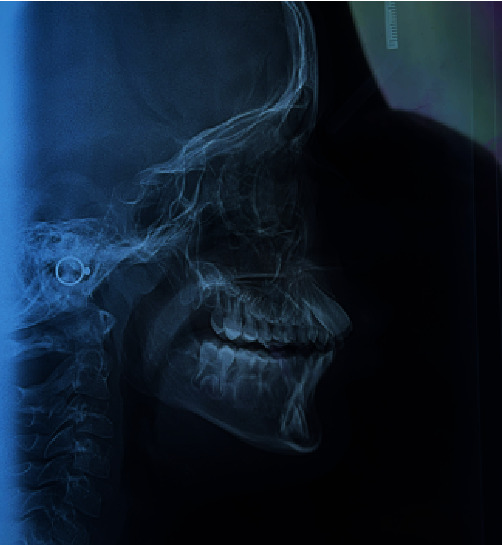
Lateral radiographic view showing skeletal class II.

The panoramic radiograph showed absence of teeth germ of teeth #15, #14, #13, #12, #22, #23, #24, #25, #35, #34, #32, #31, #41, #44, and #45. Chronologically delayed eruption and taurodontism on the first molars (Figure 2) were detected. In addition, profile radiography of the patient's skull showed skeletal class II (Figure 3).

## 4. Discussion

Oligodontia is a polygenic inheritance influenced by environmental factors and may be associated with other symptoms affecting ectodermal structures, a large group of rare diseases, such as ectodermal anhidrotic syndrome, Down syndrome, or DS [5]. Several signs of the patient are part of the clinical picture of several syndromes including fetal alcohol syndrome (FAS), Bloom syndrome (BS), and DS [6].

FAS is a condition in a child that results from alcohol exposure during the mother's pregnancy, and it causes brain damage and growth problems. The problems vary from child to child. Physical defects may include distinctive facial features (small eyes, thin upper lip, short upturned nose, small head circumference, and brain size).

Brain and central nervous system problems, heart defects, and problems with kidneys are also found in children with FAS [7]. Even if the symptoms expressed in the patient were similar to FAS, the patient's mother did not drink alcohol. BS is a rare chromosome disruption syndrome characterized by marked genetic instability associated with pre- and postnatal growth retardation, predisposition to cancer, pigmentary lesions, eye disorders, and oligodontia. Only people with BS have red and purple patches on their skin caused by blood vessels [8], a thing that was not notified in this case. All the patient's signs were closest to DS. DS was described in 1965, in a girl with intrauterine growth retardation and distinctive facies: large low set ears, retrognathia, ptosis, eczema, and short stature [6, 8]. Microcephaly, varying degrees of developmental and motoric delay, and a variety of minor anomalies, intelligence varies from severe retardation to average levels, delayed speech (60%), and hyperactivity (40%) were reported in litterature [6, 8, 9]. Aplastic anemias [6, 10], neoplasms, leukemia, lymphoma, and neuroblastoma associated with DS were reported [11]. This syndrome is associated with common physical characteristics: small stature, slow growth, microcephaly, intellectual disability, eczema, triangle shaped face, high sloping forehead, ptosis, blepharophimosis, large ears, and sparse hair [12, 13]. All these characteristics were notified in the patient besides ptosis and blepharophimosis.

DS is an autosomal recessive condition, but the specific gene mutation responsible has not yet been identified [11, 13]. It appears to affect both sexes and all ethnicities equally. The overall incidence of DS is very rare. Approximately 150 cases have been reported in the literature, with various other associated anomalies [6, 8]. Most of these cases have been reported in the USA, Europe, Middle East, Russia, and Japan [14]. This case might be the first case of DS with dental findings from the country, but a thorough examination with genetic analysis must be carried out.

In 141 individuals with DS, facial anomalies and microcephaly were present in 112 patients and blepharophimosis in 60. A prominent round nose tip was considered as especially characteristic of DS at a young age. Multiple dental carious lesions, retarded eruption, microdontia, malocclusion, diastema, and fusion of dental elements were found [6, 14]. In 1990, velopharyngeal insufficiency was described for the first time [15]. Oral features include a thin upper lip border, small oral cavity, prominent philtrum, narrow and deep palate, micrognathia, prognathism, and retrognathism [6]. The patient presented alterations of thin upper lip border, micrognathia, retarded eruption, and malocclusion.

Growth retardation, which is due to growth hormone (GH) deficiency, could be due to gene mutations [16, 17]. DS is accompanied with a deficiency in the GH, which may be caused by genetic mutations, malformations of the hypothalamus, or pituitary gland during development; GH deficiency also correlates with low levels of IgG antibodies, a condition found in Dubowitz patients [17]. DS has autosomal recessive inheritance [18]. Affected siblings have been described in nine families, with both sexes affected.

## 5. Conclusion

Oligodontia may be isolated or associated with syndromes, such as Down syndrome, BS, or DS. The diagnosis of DS is usually based on the characteristic facial appearance, growth data, and medical history. Because of the risks, early diagnosis is essential to avoid complications.

## References

[1] O’Dowling I. B., McNamara T. G. (1990). Congenital absence of permanent teeth among Irish school-children. *Journal of the Irish Dental Association*.

[2] Shimizu T., Maeda T. (2009). Prevalence and genetic basis of tooth agenesis. *Japanese Dental Science Review*.

[3] Worsaae N., Jensen B. N., Holm B., Holsko J. (2007). Treatment of severe hypodontia-oligodontia--an interdisciplinary concept. *International Journal of Oral and Maxillofacial Surgery*.

[4] Rune B., Sarnäs K. V. (1974). Tooth size and tooth formation in children with advanced hypodontia. *Angle Orthodontist*.

[5] Al-Ani A. H., Antoun J. S., Thomson W. M., Merriman T. R., Farella M. (2017). Hypodontia: an update on its etiology, classification, and clinical management. *BioMed Research International*.

[6] Dubowitz V. (1965). Familial low birthweight dwarfism with unusual facies and a skin eruption. *Journal of Medical Genetics*.

[7] Mathieu M., Berquin P., Epelbaum S., Lenaerts C., Piussan C. (1991). Le syndrome de Dubowitz. Un diagnostic à ne pas méconnaître [Dubowitz syndrome. A diagnosis not to be missed]. *Archives Francaises de Pediatrie*.

[8] Winter R. M. (1986). Dubowitz syndrome. *Journal of Medical Genetics*.

[9] Moller K. T., Gorlin R. J. (1985). The Dubowitz syndrome: a retrospective. *Journal of Craniofacial Genetics and Developmental Biology Supplement*.

[10] Opitz J. M., Pfeiffer R. A., Hermann J. P., Kushnick T. (1973). Studies of malformation syndromes of man XXIV B: the Dubowitz syndrome. Further observations. *Zeitschrift fur Kinderheilkunde*.

[11] Modell B., Darlison M. W., Malherbe H. (2018). Congenital disorders: epidemiological methods for answering calls for action. *Journal of Community Genetics*.

[12] Kondo I., Takeda K., Kuwajima K., Hirano T. (1987). A Japanese patient with the Dubowitz syndrome. *Clinical Genetics*.

[13] Ballini A., Cantore S., Tullo D., Desiate A. (2011). Dental and craniofacial characteristics in a patient with Dubowitz syndrome: a case report. *Journal of Medical Case Reports*.

[14] Hamosh A., Scott A. F., Amberger J., Valle D., McKusick V. A. (2000). Online Mendelian inheritance in man (OMIM). *Human Mutation*.

[15] Ahmad A., Amalfitano A., Chen Y. T., Kishnani P. S., Miller C., Kelley R. (1999). Dubowitz syndrome: a defect in the cholesterol biosynthetic pathway?. *American Journal of Medical Genetics*.

[16] Lyonnet S., Schwartz G., Gatin G., de Prost Y., Munnich A., Le Merrer M. (1992). Blepharophimosis, eczema, and growth and developmental delay in a young adult: late features of Dubowitz syndrome?. *Journal of Medical Genetics*.

[17] Hirano T., Izumi I., Tamura K. (1996). Growth hormone deficiency in Dubowitz syndrome. *Acta Paediatrica Japonica*.

[18] Lerman-Sagie T., Merlob P., Shuper A. (1990). New findings in a patient with Dubowitz syndrome: velopharyngeal insufficiency and hypoparathyroidism. *American Journal of Medical Genetics*.

